# Beneficial Effects of Remimazolam Compared with Dexmedetomidine as an Adjuvant in Total Intravenous Anaesthesia with Propofol and Remifentanil: A Randomised Controlled Trial

**DOI:** 10.3390/medicina62020303

**Published:** 2026-02-02

**Authors:** Seung-Wan Hong, Seong-Hyop Kim

**Affiliations:** 1 Department of Anesthesiology and Pain Medicine, Konkuk University Medical Center, Konkuk University School of Medicine, Seoul 05030, Republic of Korea; 2 Department of Infection and Immunology, Konkuk University School of Medicine, Seoul 05030, Republic of Korea; 3 Department of Medicine, Institute of Biomedical Science and Technology, Konkuk University School of Medicine, Seoul 05030, Republic of Korea; 4 Department of Medical Education, Konkuk University School of Medicine, Seoul 05030, Republic of Korea

**Keywords:** total intravenous anaesthesia, propofol, remimazolam, dexmedetomidine, haemodynamics, postoperative nausea and vomiting

## Abstract

*Background and Objectives:* The study was designed to compare the propofol-sparing effect, intraoperative haemodynamic profiles, and recovery profiles during propofol–remifentanil total intravenous anaesthesia (TIVA) with remimazolam or dexmedetomidine co-administered as an adjuvant. *Materials and Methods:* After the remifentanil target concentration of 5 ng/mL had been achieved and endotracheal intubation was completed, the R group was intravenously administered 1 mg remimazolam/kg/hr, the D group was given 0.5 µg dexmedetomidine/kg/hr, and the control (C) group was given 1 mL normal saline/kg/hr. The allocated experimental drug infusion was initiated immediately after intubation and maintained until termination of the two target-controlled infusions of propofol and remifentanil. The propofol-sparing effect, intraoperative haemodynamic profiles and recovery profiles were assessed in the three groups. *Results:* The R group had the lowest requirement of propofol and the C group had the highest requirement of propofol. Haemodynamic profiles were similar among the groups. However, the total phenylephrine dose administered to maintain haemodynamic stability was significantly lower in the R group than in the D group and C group. Recovery profiles did not significantly differ between the groups. *Conclusions:* The co-administration of remimazolam or dexmedetomidine as an adjuvant in propofol–remifentanil TIVA reduced propofol requirements. While recovery profiles, including recovery times, postoperative pain, and postoperative nausea and vomiting, were similar among the groups, remimazolam was associated with a reduced phenylephrine requirement despite similar haemodynamic profiles.

## 1. Introduction

Balanced anaesthesia was introduced by John Lundy in 1926 [1,2], with the aim of providing safe general anaesthesia while minimising the adverse effects of any single agent by using two or more drugs in combination rather than one alone.

Total intravenous anaesthesia (TIVA) has become a cornerstone of modern anaesthetic practice because of its advantages, including rapid onset and offset, reduced postoperative nausea and vomiting, and avoidance of volatile anaesthetic-related side effects. Propofol, a widely used intravenous anaesthetic, typically forms the basis of TIVA owing to its favourable pharmacokinetic profile. However, its use is often associated with dose-dependent hypotension and other concerns, such as the rare but serious propofol infusion syndrome, particularly during prolonged infusions [3]. To mitigate these risks and enhance haemodynamic stability, adjuvant drugs are frequently co-administered with propofol [4,5,6].

Remimazolam, a novel ultra-short-acting benzodiazepine, represents a promising adjuvant because of its rapid and predictable metabolism via tissue esterases, leading to a quick recovery profile and minimal cardiovascular depression. Its mechanism of action primarily involves modulation of GABA_A receptors, enhancing inhibitory neurotransmission [7]. Given these characteristics, remimazolam may reduce propofol requirements while maintaining or improving intraoperative haemodynamic stability.

Dexmedetomidine, an α2-adrenergic agonist, is another established adjuvant in TIVA and is well regarded for its sedative and analgesic properties as well as its significant propofol-sparing effects [8,9].

The distinct pharmacological profiles of remimazolam and dexmedetomidine suggest that they may exert differential effects on propofol requirements, haemodynamics, and recovery following TIVA. While both agents can reduce propofol dose, their unique mechanisms could lead to different clinical outcomes. Therefore, this study was designed to evaluate and compare the propofol-sparing effect of remimazolam versus dexmedetomidine, as well as their respective influences on intraoperative haemodynamics and recovery profiles, when used as adjuvants to propofol–remifentanil-based TIVA.

## 2. Materials and Methods

### 2.1. Study Population

The study was approved by the Institutional Review Board of Konkuk University Medical Center, Seoul, Korea (reference number, KUH 2021-10-038; approval date, 19 May 2022) and informed consent was obtained from all patients. All procedures involving human participants were in accordance with the ethical standards of the institutional and national research committee and with the 1975 Helsinki Declaration, as revised in 2013. The study was registered at the Clinical Research Information Service, Korea Centers for Disease Control and Prevention, Ministry of Health and Welfare (KCT0007473; registration date, 30 June 2022; http://cris.nih.go.kr) (accessed on 27 January 2026). The registry record was updated during revision to correct a selection/data-entry error in the intervention model classification (factorial to parallel) and to clarify prespecified secondary outcomes, including total intraoperative vasopressor requirement (including phenylephrine).

Non-smoking female patients without significant medical history (American Society of Anesthesiologists physical status I) who were scheduled for elective breast surgery under remifentanil-based anaesthesia were eligible. The exclusion criteria were urgent or emergent cases, allergy to egg or soybean oil, QT prolongation in preoperative electrocardiography, history of drug abuse, any regular medications that could materially affect haemodynamic responses or anaesthetic requirements (e.g., antihypertensive agents, beta-blockers, or antiarrhythmics), other concurrent surgery, and use of a nerve block or intravenous patient-controlled analgesia. Before inducing anaesthesia, the patients were randomly allocated in a 1:1:1 ratio to the R, D, or C group by opening a sequentially numbered, sealed envelope containing the randomisation assignment. The allocation sequence was generated by an anaesthesiologist who was not involved in patient recruitment or intraoperative management, using random-permuted block randomisation. The attending anaesthesiologists and patients remained blinded to group allocation throughout the study. Postoperative outcomes (VAS and PONV) and other study data were collected by trained observers who did not participate in patient care and were blinded to group allocation.

### 2.2. Remifentanil-Based Anaesthesia

The anaesthetic technique was standardised. Patients arrived at the operating room without premedication. Anaesthesia was induced after routine non-invasive patient monitoring, including pulse oximetry, electrocardiography, non-invasive blood pressure (NIBP) and bispectral index (BIS) monitoring. The anaesthesiologists were blinded to the study and anaesthetised the patients as described below. Lidocaine (0.5 mg/kg) was administered to decrease propofol-related pain. The anaesthetics were intravenously administered using two target-controlled infusion (TCI) devices, with an initial propofol concentration of 3 µg/mL for effect-site targeted infusion according to the modified Marsh model (*k_e_*_0_ of 1.21/min) [10]. No fixed propofol bolus was administered and induction was performed using effect-site targeted TCI starting at 3.0 μg/mL, followed by BIS-guided titration according to the study protocol as described below. Remifentanil was administered via effect-site target-controlled infusion using the Minto model, with a fixed effect-site target concentration (Ce) of 5 ng/mL [11]. The remifentanil target concentration of 5 ng/mL was achieved within 3 min after drug administration and maintained during anaesthesia. During maintenance of anaesthesia, effect-site concentration of propofol was titrated to maintain BIS values between 50 and 60. If BIS values exceeded 60, the propofol target concentration was increased by 0.5 μg/mL increments within 60 s. If BIS remained above 60 after 5 min, the concentration was further increased by 0.5 μg/mL every 5 min. Conversely, if BIS dropped below 50, the propofol target concentration was decreased by 0.5 μg/mL within 60 s, with further decrements every 5 min if BIS remained below 50. The minimum propofol target concentration was set at 2.5 μg/mL. BIS was recorded at 5 min intervals. After the patient had lost consciousness, rocuronium (0.6 mg/kg) was administered for muscle relaxation under the guidance of peripheral neuromuscular transmission (NMT) monitoring. Endotracheal intubation was performed after the remifentanil effect-site concentration of 5 ng/mL was achieved and the train-of-four count reached 0. After anaesthesia induction, the patient was ventilated with 40% oxygen in air. The tidal volume was 6 mL/kg ideal body weight, with a positive end-expiratory pressure of 5 cm H_2_O. The respiratory rate was adjusted to keep the partial pressure of end-tidal carbon dioxide between 35 and 40 mmHg at an inspiration/expiration ratio of 1:2. Additional rocuronium was administered under the guidance of peripheral NMT monitoring. As previously described [12], hypotension or bradycardia was managed using phenylephrine (30 µg) when the mean arterial pressure (MAP; non-invasive oscillometric value) was <60 mmHg with a heart rate (HR) ≥ 40 beats/min, ephedrine (4 mg) when MAP was <60 mmHg with HR < 40 beats/min, and atropine (0.5 mg) when HR was <40 beats/min. For patients with MAP < 60 mmHg, repetitive bolus injections of 30 µg phenylephrine were administered every 60 s. If MAP remained < 60 mmHg despite these bolus injections, phenylephrine was initiated as a continuous infusion at a rate of 0.2 µg/kg/min and titrated to maintain MAP ≥ 60 mmHg. Patients with a systolic blood pressure > 180 mmHg or a diastolic blood pressure > 110 mmHg were administered 0.5 mg nicardipine, and those with an MAP > 60 mmHg and a HR > 110 beats/min were administered 30 mg esmolol during anaesthesia after the remifentanil target concentration of 5 ng/mL had been reached. TCIs of propofol and remifentanil were stopped after completion of surgery and application of dressings. Intravenous ketorolac (0.5 mg/kg) was administered for postoperative pain control, and ramosetron (0.3 mg) was given intravenously to prevent postoperative nausea and vomiting (PONV). Residual neuromuscular paralysis was antagonised with 2 mg sugammadex/kg under the guidance of peripheral NMT monitoring. After endotracheal extubation, the patient was transferred to the post-anaesthetic care unit (PACU). The standardised anaesthetic sequence is summarised in Supplementary Figure S1 for clarity and reproducibility.

### 2.3. Remimazolam and Dexmedetomidine as an Adjuvant for Anaesthesia

Remimazolam, dexmedetomidine, and normal saline (hereafter referred to as the experimental drugs) were prepared for each patient by an independent registered nurse according to the concealed allocation sequence. The nurse was not involved in patient care, outcome assessment, or data analysis, and identical syringes were used to maintain blinding. Patients in the R, D and C groups were administered 1 mg/kg/hr of remimazolam, 0.5 µg/kg/hr of dexmedetomidine, and 1 mL/kg/hr of saline, respectively. For the R group, the syringe contained remimazolam at a concentration of 1 mg/mL, prepared by dissolving 50 mg of remimazolam (Byfavo™, Hana Pharm Co., Ltd., Seoul, Republic of Korea) in 50 mL of normal saline. For the D group, the syringe contained dexmedetomidine at a concentration of 0.5 µg/mL, prepared by adding a 50 mL premixed solution containing 200 µg dexmedetomidine (Precedex^®^, Pfizer Inc., New York, NY, USA; 4 µg/mL) to 350 mL of normal saline, from which 50 mL was drawn for administration. For the C group, the syringe contained 50 mL of normal saline.

After the remifentanil effect-site target concentration of 5 ng/mL had been achieved and endotracheal intubation was completed, the allocated experimental drug infusion was initiated immediately after intubation (before skin incision) and maintained until termination of the target-controlled infusions of propofol and remifentanil.

### 2.4. Measurements

Anaesthesia time, defined as the time from anaesthesia induction until discharge from the operating room, and operation time, defined as beginning with the surgical incision and ending with skin dressing, were recorded.

The propofol-sparing effect was assessed by measuring the infused amounts of propofol, remifentanil, and the experimental drugs administered to each group during anaesthesia.

Intraoperative haemodynamic profiles were assessed based on MAP, HR, and BIS, measured just before the start of experimental drugs (T_start_); at 30 min (T_30 min_) and 60 min (T_60 min_) after the start of experimental drugs; at the termination of experimental drugs (T_stop_); and just after endotracheal extubation (T_extubation_). The total dose of each administered vasoactive agent was checked during anaesthesia.

For recovery profiles, recovery times (from the termination of all infused anaesthetics to the first BIS value > 80 during emergence from anaesthesia [BIS_80_] and from the termination of all infused anaesthetics to endotracheal extubation [extubation]) were recorded. MAP and HR were measured from the time of PACU arrival (T_PACUin_) until PACU discharge (T_PACUout_). Postoperative pain was evaluated using a visual analogue scale (VAS) ranging from 0 (no pain) to 100 (the worst pain imaginable), and PONV using a four-point ordinal scale (0 = none, 1 = nausea, 2 = retching, 3 = vomiting). The maximum VAS and PONV scores during the PACU stay were recorded. VAS scores were recorded at 24 h and 48 h postoperatively. PONV severity at 24 h and 48 h was additionally assessed using the Rhodes Index. The index uses a numerical scale from 0 to 32, including subjective (degree of severity) and objective (with/without nausea, retching, and vomiting, and times of nausea, retching, and vomiting) aspects of PONV [13,14]. The first rescue for postoperative pain was ketorolac, administered intravenously on demand at a dose of 0.5 mg/kg. The second rescue for postoperative pain was fentanyl, administered intravenously on demand at a dose of 0.2 µg/kg. The first rescue for PONV was dexamethasone and the second was ondansetron, administered intravenously on demand at doses of 5 mg and 4 mg, respectively.

### 2.5. Statistics

The primary outcome was total propofol requirement. Secondary outcomes were the total dose of administered phenylephrine and the recovery time, defined as the time to endotracheal extubation. Effect sizes for these outcomes were estimated from a preliminary pilot study including 15 patients (5 per group). Partial eta-squared (η_p_^2^) values for between-group differences in propofol requirement, total phenylephrine dose, and recovery time were obtained from one-way ANOVA and transformed into Cohen’s f using the standard formula f = √[η_p_^2^/(1 − η_p_^2^)]. The resulting effect sizes (propofol requirement, f = 0.67; phenylephrine dose, f = 1.07; recovery time, f = 0.50) were entered into G*Power version 3.1.9.7 (Heinrich-Heine-Universität Düsseldorf, Düsseldorf, Germany) with the following settings: test family = F tests; statistical test = ANOVA: fixed effects, omnibus, one-way; α error probability = 0.05; power (1 − β) = 0.90; and number of groups = 3. The required total sample sizes were 33 for propofol requirement, 15 for phenylephrine dose, and 54 for recovery time [15,16]. The final target sample size was therefore set at 54, based on the largest requirement among the primary and key secondary outcomes. To allow for an anticipated dropout rate of approximately 10%, institutional review board (IRB) approval was obtained for the enrolment of up to 60 patients (20 per group). Recruitment was planned to cease once 54 analysable participants (18 per group) had been enrolled, in accordance with the protocol and sample size calculation. Pilot data were used exclusively for sample size estimation and were not included in the final analysis set.

Statistical analyses were performed using the Statistical Package for the Social Sciences (SPSS) for Windows, version 27.0 (SPSS, Chicago, IL, USA). Continuous variables were expressed as mean ± standard deviation or median [interquartile range], and categorical variables as numbers and percentages. A *p*-value of less than 0.05 was considered statistically significant. The normality of continuous variables was assessed using the Shapiro–Wilk test. For variables that satisfied the normality assumption, between-group comparisons were performed using one-way ANOVA; otherwise, the Kruskal–Wallis test was applied. When overall group differences were significant, post hoc pairwise comparisons were conducted with Bonferroni adjustment for multiple testing. Intergroup and intragroup changes over time (e.g., MAP, HR, and BIS values) were evaluated using two-way and one-way repeated-measures ANOVA, respectively, after confirming normality. The assumption of sphericity was examined with Mauchly’s test, and, when violated, the Greenhouse–Geisser correction was applied. Post hoc pairwise comparisons following significant repeated-measures ANOVA results were performed with Bonferroni adjustment. Group differences in incidence data (categorical variables) were assessed using the chi-square test or Fisher’s exact test, as appropriate. For key outcomes, effect size estimates with corresponding 95% confidence intervals were additionally reported to aid clinical interpretation.

## 3. Results

**Figure 1:**
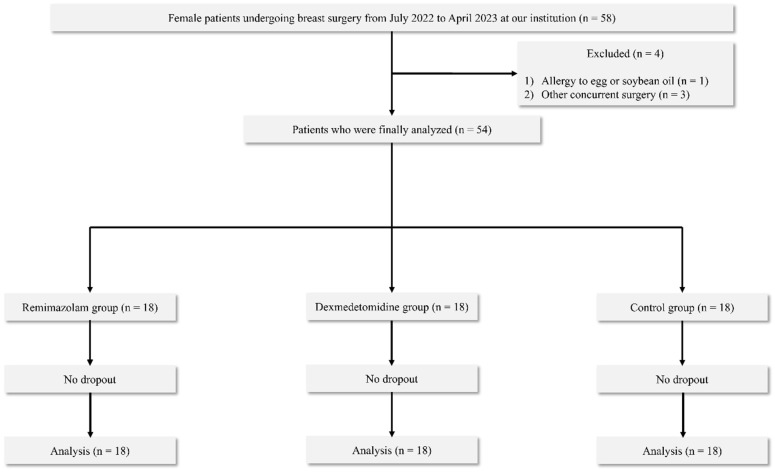
Flow of the participants for the study.

Out of 58 patients who underwent initial screening, 4 were excluded based on our prespecified criteria (1 for egg allergy, 3 for concurrent surgeries). This resulted in a cohort of 54 patients (18 per group) who were then randomised, successfully completed the study without any loss to follow-up, and were included in the final analysis (Figure 1). Although the total enrolment did not reach 60, recruitment was stopped as planned after achieving the target sample size of 54 analysable participants, which satisfied the statistical power requirements outlined in our protocol. Baseline demographic characteristics were comparable among the three groups, with all standardised mean differences (SMDs) ≤ 0.2 (Table 1). Effect size estimates regarding key outcomes are presented in Supplementary Table S1. Because several continuous variables did not meet normality assumptions, non-parametric analyses were applied where appropriate, as specified in the Statistical Analysis section.

The propofol dose was significantly lower in both the R group (274.5 [231–358.8] mg) and the D group (335.5 [305.5–503.8] mg) compared to the C group (619 [514.3–940] mg; R and C *p* < 0.001, D and C *p* = 0.002). However, there was no significant difference in propofol dose between the R and D groups (*p* = 0.231) (Table 2).

**Figure 2:**
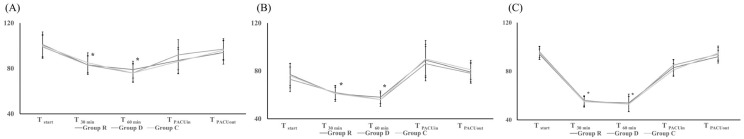
Haemodynamic parameters including mean arterial pressure (MAP) (**A**), heart rate (HR) (**B**) and bispectral index (BIS) (**C**) during anaesthesia and post-anaesthetic care unit (PACU) stay (except BIS during PACU stay). Abbreviations: T_start_, just before the start of experimental drugs; T_30 min_ and T_60 min_, at 30 min and 60 min after the start of experimental drugs, respectively; T_PACUin_, on arrival at PACU; T_PACUout_, at discharge from PACU. * *p* < 0.05 after Bonferroni correction compared with T_start_.

In all three groups, MAP, HR, and BIS followed similar patterns, decreasing after the start of remimazolam (R group), dexmedetomidine (D group), or normal saline (C group) but then recovering after ceasing their use (Figure 2). The intergroup differences in MAP, HR, and BIS were not significant (Figure 2). The total dose of phenylephrine administered for haemodynamic stability during anaesthesia was significantly lower in the R group (385 [0–430]) than in the D (627.5 [226.3–955]; *p* = 0.042) or C (795 [258.8–1232.5]; *p* = 0.009) groups. The difference between the D and C groups was not significant (Table 2).

Recovery time, including BIS_80_ (5 [4,5,6] min in the R group vs. 6 [4,5,6,7] in the D group vs. 5 [4,5,6] in the C group; *p* = 0.192) and extubation (7 [6,7] min in the R group vs. 7 [6–8.3] min in the D group vs. 7 [6,7] min in the C group; *p* = 0.179), did not significantly differ between the three groups (Table 3). There were also no significant differences between groups with respect to MAP and HR during the PACU stay (Figure 2). Postoperative pain and PONV according to the Rhodes Index at 24 and 48 h postoperatively were also similar between the groups (*p* > 0.05) (Table 3).

## 4. Discussion

The co-administration of either remimazolam or dexmedetomidine as an adjuvant in TIVA with propofol and remifentanil reduced the propofol requirement, with both remimazolam and dexmedetomidine co-administration significantly lowering the propofol dose compared to the control group. When co-administered as an adjuvant during propofol–remifentanil TIVA, remimazolam was associated with a significantly lower phenylephrine requirement than dexmedetomidine or saline, despite similar haemodynamic profiles among the groups. However, recovery profiles, including recovery times, postoperative pain and PONV, did not significantly differ between the three groups.

Both remimazolam and dexmedetomidine have sedative effects, which may explain the reduced propofol requirement in the R and D groups. The propofol requirement was lowest in the R group. This might be attributed to remimazolam’s known capability for anaesthesia induction and maintenance even as a sole agent, a characteristic not shared by dexmedetomidine.

The use of an intravenous anaesthetic agent for sedation or amnesia, particularly propofol, often causes a decrease in blood pressure, attributable to decreases in systemic vascular resistance and cardiac output, with unfavourable or catastrophic results. In this study, the total dose of phenylephrine administered for haemodynamic stability during anaesthesia was significantly lower in the R group than in the D and C groups. The haemodynamic stability achieved with remimazolam was previously reported [17,18]. Better haemodynamic stability than other intravenous anaesthetic agents, except ketamine, is a characteristic of ester-based benzodiazepines. Frölich et al. compared the haemodynamic effects of midazolam, dexmedetomidine, and propofol and found dose-dependent reductions in arterial blood pressure following dexmedetomidine and propofol administration, whereas the effect of midazolam on blood pressure was minimal [19]. Lim et al. also reported less haemodynamic suppression at anaesthesia induction following the co-administration of midazolam and propofol than propofol alone [20]. Urabe et al. found that the haemodynamic stability achieved with remimazolam was associated with the calcium concentration in endothelial and neuronal cells via the G-protein coupled receptors-inositol 1,4,5-triphosphate pathway [21].

In the D group, the total dose of phenylephrine required to maintain haemodynamic stability did not differ significantly from that in the C group. Previous studies have reported that co-administered dexmedetomidine in TIVA with propofol and remifentanil was associated with a lower MAP and HR and significantly fewer fluctuations in haemodynamic parameters [22]. Therefore, in the D group, there was no benefit in any aspect of haemodynamic stability, but the propofol requirement was significantly reduced.

Although propofol allows a rapid emergence from anaesthesia, due to its short context-sensitive half-time, propofol use over a longer duration results in delayed emergence. In the present study, the co-administration of remimazolam or dexmedetomidine reduced the propofol requirement, but it did not lead to an earlier recovery than in the C group. Nevertheless, reducing overall propofol exposure may still be clinically meaningful given its dose-dependent cardiovascular depression. Therefore, adjuvant co-administration may be beneficial in patients susceptible to haemodynamic instability, even when postoperative recovery profiles are comparable. The rapid onset and offset characteristics of remimazolam were expected to allow a rapid emergence from anaesthesia. However, the recovery time following sedation or anaesthesia was longer with either remimazolam alone or dexmedetomidine alone than with propofol alone [23,24,25]. This can be explained by the fact that remimazolam (R group) and dexmedetomidine (D group) were co-administered without a loading dose, which might have limited the effects of these drugs. In all three groups, the anaesthesia time was about 120 min. A longer time would likely have yielded different recovery times. In addition, the high effect-site concentration of remifentanil (5 ng/mL) meant that TIVA was opioid (remifentanil)-based anaesthesia. Hypnotic (propofol)-based anaesthesia would have followed from a higher propofol concentration and a lower remifentanil concentration and might have yielded different results. More remarkable effects in hypnotic-based than in opioid-based anaesthesia would likely have been obtained by the co-administration of remimazolam or dexmedetomidine as an adjuvant. To address the delayed recovery associated with remimazolam use, the benzodiazepine antagonist flumazenil is administered. Whether flumazenil can be safely used as a remimazolam antagonist is unclear, and adverse effects have been reported [26,27].

In the present study, the propofol, remimazolam, and dexmedetomidine doses were adjusted, using BIS, after fixation of the target concentration for remifentanil. Thus, three intravenous anaesthetic agents, propofol, remimazolam, and dexmedetomidine, were used to achieve sedation or amnesia. However, the analgesic effect of dexmedetomidine, in contrast to that of propofol and remimazolam, might have been limited due to the use of a higher remifentanil concentration.

While BIS is primarily validated for monitoring propofol-induced hypnotic states, accumulating evidence suggests its utility in assessing the depth of sedation and anaesthesia with benzodiazepines and α2 agonists. Studies have demonstrated that BIS values correlate with the sedative effects of both remimazolam and dexmedetomidine, reflecting changes in cortical electrical activity [28,29,30]. Although their specific electroencephalogram (EEG) signatures may differ from propofol, BIS provides a practical and widely used clinical tool for titrating overall anaesthetic depth, especially in multi-agent TIVA. Our study’s reliance on BIS for propofol titration, while acknowledging the influence of other agents, is thus clinically justified as it provides a standardised objective measure of central nervous system depression across all groups.

In this study, we evaluated remimazolam and dexmedetomidine regarding anaesthesia induction, maintenance, and emergence profiles when co-administered with propofol and remifentanil in TIVA. This approach differs from previous studies that have evaluated these agents individually [31,32]. This study specifically evaluated the propofol requirement during anaesthesia induction and maintenance, the phenylephrine requirement for anaesthesia maintenance and the recovery profiles, defined as anaesthesia emergence.

This study has several limitations. First, the sample size was modest (18 participants per group), which may limit the precision of between-group comparisons. Second, this was a single-centre study restricted to non-smoking female patients undergoing breast surgery; thus, external validity to other surgical populations, male patients, or higher-risk cohorts may be limited.

## 5. Conclusions

In propofol–remifentanil TIVA, the co-administration of remimazolam or dexmedetomidine as an adjuvant reduced propofol requirements. Recovery profiles, including recovery times, postoperative pain, and postoperative nausea and vomiting, were similar among the groups. However, remimazolam was associated with a reduced phenylephrine requirement despite similar haemodynamic profiles. Therefore, remimazolam may be a useful adjuvant in propofol–remifentanil TIVA. These findings should be interpreted cautiously, given the modest sample size and the selected study population.

## Figures and Tables

**Table 1:** Demographic data.

				Standardised Mean Differences		
	R Group (*n* = 18)	D Group (*n* = 18)	C Group (*n* = 18)	R Group vs. D Group	R Group vs. C Group	D Group vs. C Group
Age (years)	53 ± 15	54 ± 13	53 ± 11	0.07	0.00	0.08
Height (cm)	158.7 ± 4.5	157.9 ± 6.6	158.1 ± 5.3	0.14	0.12	0.03
Weight (kg)	55.5 [50.4–63.5]	56.0 [50.0–65.5]	54.5 [51.2–63.3]	0.10	0.05	0.05
Hx of motion sickness	2/18	2/18	1/18	0.00	0.20	0.20
Hx of PONV	1/18	1/18	2/18	0.00	0.20	0.20
Anaesthesia time (min)	122.5 [101.5–149.8]	122.5 [93.8–147.5]	120 [109–153.8]	0.09	0.03	0.12
Operation time (min)	99.5 [76.5–123.3]	97.5 [67.5–121.8]	96 [82.5–125]	0.11	0.00	0.11

Data are expressed as the number of patients, mean ± standard deviation or median [first–third quartiles]. Abbreviations: Hx, history; PONV, postoperative nausea and vomiting.

**Table 2:** Intraoperative profiles.

		R Group (*n* = 18)	D Group (*n* = 18)	C Group (*n* = 18)	*p* Value
Anaesthetic agents					
	Propofol (mg) *	274.5 [231–358.8]	335.5 [305.5–503.8]	619 [514.3–940]	<0.001
	Remifentanil (μg)	1160 [970.5–1465.8]	1106 [815.3–1541.3]	1282 [1156.3–1838]	0.192
	Rocuronium (mg)	34 [30.8–38.3]	34 [30.5–40.3]	33 [31–38.3]	0.956
	Remimazolam (mg)	115 [94.5–141.5]	-	-	-
	Dexmedetomidine (μg)	-	55 [41–87.3]	-	-
	Normal saline (mL)	-	-	109.5 [99.5–144.5]	-
Vasoactive agents					
	Phenylephrine (μg) ^†^	385 [0–430]	627.5 [226.3–955]	795 [258.8–1232.5]	0.007
	Ephedrine (mg)	-	-	-	-
	Atropine (mg)	-	-	-	-
	Nicardipine (mg)	-	-	-	-
	Esmolol (mg)	-	-	-	-

Data are expressed as median [first–third quartiles]. * Overall *p*-values were obtained using the Kruskal–Wallis test. Post hoc pairwise comparisons were performed with Bonferroni-adjusted *p* values: R group vs. D group (*p* = 0.231); D group vs. C group (*p* = 0.002); R group vs. C group (*p* < 0.001). ^†^ Overall *p*-values were obtained using the Kruskal–Wallis test. Post hoc pairwise comparisons were performed with Bonferroni-adjusted *p* values: R group vs. D group (*p* = 0.042); D group vs. C group (*p* = 1.000, not significant); R group vs. C group (*p* = 0.009).

**Table 3:** Postoperative profiles.

		R Group (*n* = 18)	D Group (*n* = 18)	C Group (*n* = 18)	*p* Value
Recovery time					
	BIS_80_ (min)	5 [4–6]	6 [4–7]	5 [4–6]	0.192
	Extubation (min)	7 [6–7]	7 [6–8.3]	7 [6–7]	0.179
Postoperative pain (0–100)					
	VAS during PACU stay	40 [27.5–40]	35 [30–40]	30 [27.5–40]	0.682
	VAS at postoperative 24 h	30 [20–30]	20 [20–30]	20 [17.5–30]	0.207
	VAS at postoperative 48 h	20 [10–30]	20 [17.5–30]	20 [10–20]	0.667
Incidence of rescue for postoperative pain	min				
	Ketorolac	10/18	11/18	8/18	0.594
	Fentanyl	3/18	2/18	4/18	0.670
PONV incidence & scale (0–3)					
	During PACU stay	1/18 & 0 [0–0]	2/18 & 0 [0–0]	1/18 & 0 [0–0]	0.763 & 0.767
	At postoperative 24 h	4/18 & 0 [0–0.3]	3/18 & 0 [0–0]	5/18 & 0 [0–1]	0.725 & 0.806
	At postoperative 48 h	0/18 & 0 [0–0]	1/18 & 0 [0–0]	2/18 & 0 [0–0]	0.347 & 0.156
Rhodes index (0–32)					
	At postoperative 24 h	0 [0–0]	0 [0–0]	0 [0–3]	0.817
	At postoperative 48 h	0 [0–0]	0 [0–0]	0 [0–0]	0.152
Incidence of rescue for PONV					
	Dexamethasone	1/18	2/18	1/18	0.763
	Ondansetron	1/18	0/18	0/18	0.361

Data are expressed as the number of patients, median [first–third quartiles]. Abbreviations: BIS, bispectral index; BIS_80_, from the stop of all infused anaesthetics to the first BIS value above 80 during emergence from anaesthesia; Extubation, from the stop of all infused anaesthetics to endotracheal extubation; VAS, visual analogue pain scale; PACU, post-anaesthetic care unit; PONV, postoperative nausea and vomiting.

## Data Availability

The datasets generated and analysed during the current study are available from the corresponding author on reasonable request.

## Supplementary Materials

The following supporting information can be downloaded at: https://www.mdpi.com/article/10.3390/medicina62020303/s1, Figure S1. Standardised Anaesthetic Protocol; Figure S2. Assessment Timeline; Table S1. Effect size estimates with 95% confidence intervals for key outcomes.
