# Supplementary material for: Beneficial Effects of Remimazolam Compared with Dexmedetomidine as an Adjuvant in Total Intravenous Anaesthesia with Propofol and Remifentanil: A Randomised Controlled Trial
Source: Medicina (Kaunas). 2026 Feb 2;62(2):303. doi: 10.3390/medicina62020303 (PMC12941681; doi:10.3390/medicina62020303)
Supplement: Supplementary file 1 [file medicina-62-00303-s001.zip › Supplementary Table S1.pdf]

Supplementary table S1. Effect size estimates with 95% confidence intervals for key outcomes

Outcome	Comparison	Hodges-Lehmann median difference	95% CI [lower – upper]	Adjusted *P* value
Total Propofol requirement (mg)				
	R vs D	81.3	27.0 – 151.8	0.231
	D vs C	262.5	159.0 – 447.0	0.002
	R vs C	342.5	246.0 – 525.0	< 0.001
Total Phenylephrine dose (μg)				
	R vs D	305.0	100.0 – 600.0	0.042
	D vs C	162.5	-245.0 – 540.0	1.000
	R vs C	480.0	145.0 – 825.0	0.009
Time to extubation (min)				
	R vs D	1.0	0.0 – 1.0	0.542
	D vs C	-1.0	-2.0 – 0.0	0.224
	R vs C	0.0	-1.0 – 0.0	1.000
