# Supplementary figures and images for: Beneficial Effects of Remimazolam Compared with Dexmedetomidine as an Adjuvant in Total Intravenous Anaesthesia with Propofol and Remifentanil: A Randomised Controlled Trial
Source: Medicina (Kaunas). 2026 Feb 2;62(2):303. doi: 10.3390/medicina62020303 (PMC12941681; doi:10.3390/medicina62020303)

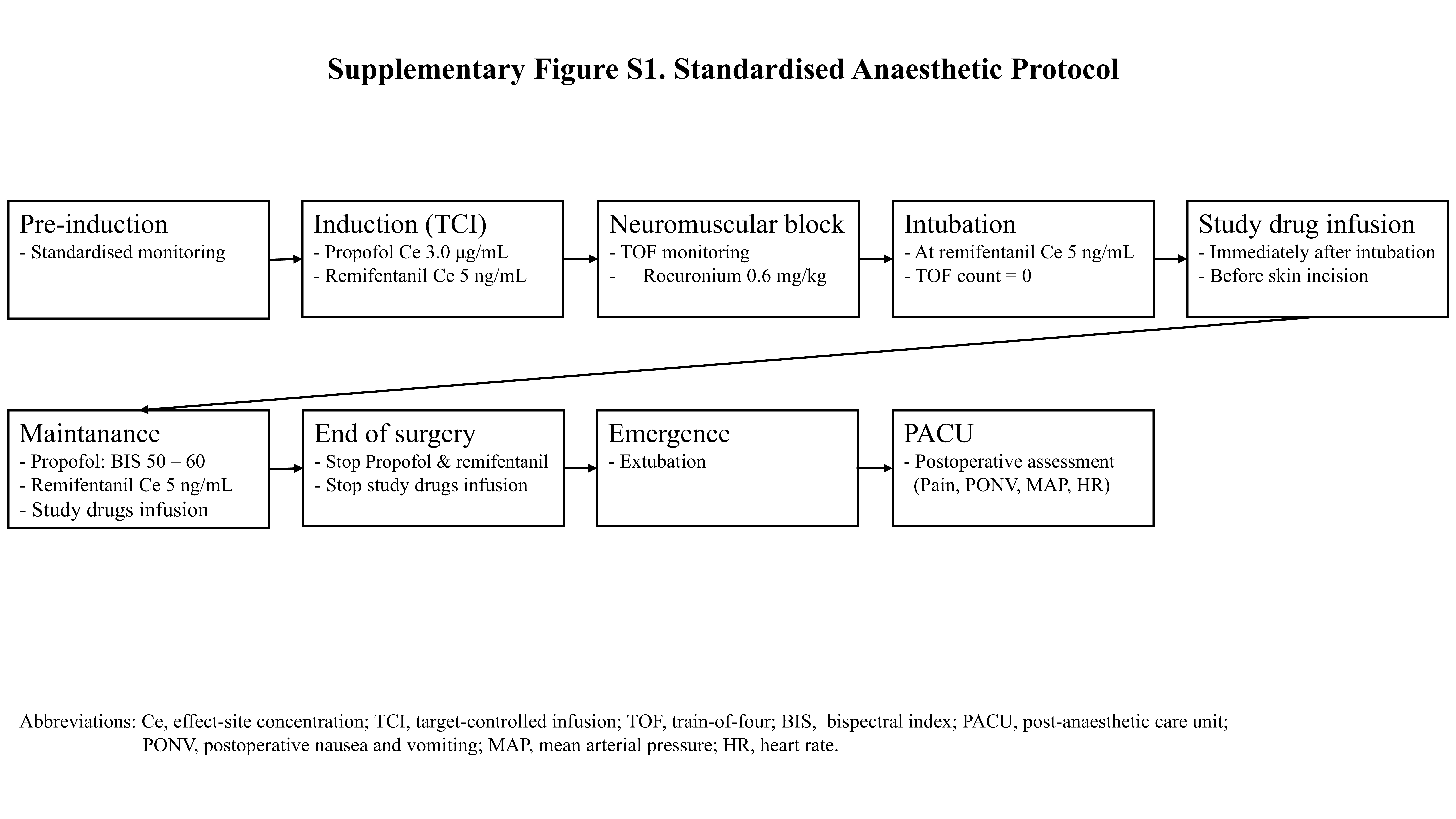

Supplement: Supplementary file 1 [file medicina-62-00303-s001.zip › Figure S1.TIF]

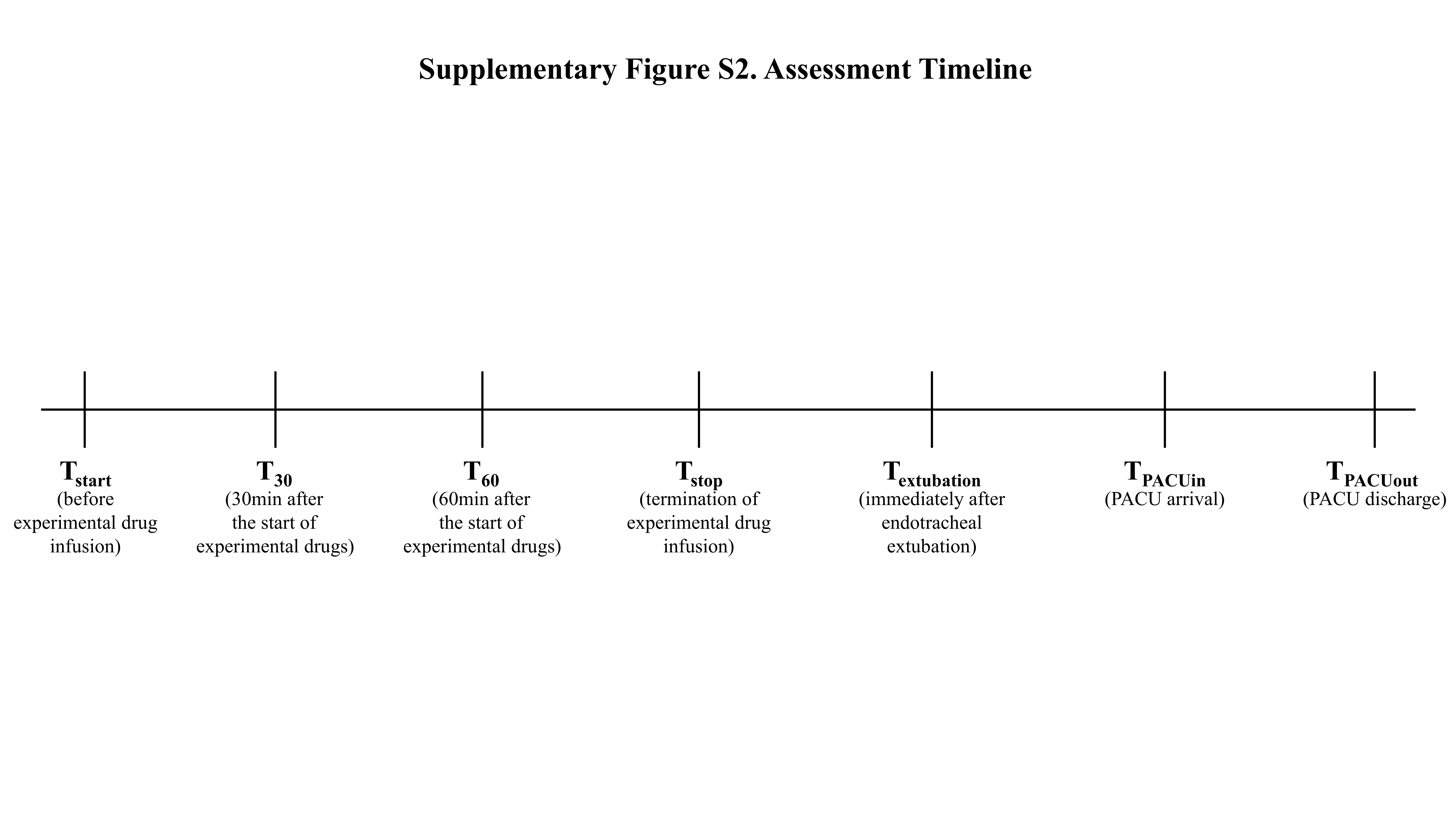

Supplement: Supplementary file 1 [file medicina-62-00303-s001.zip › Figure S2.TIF]
